# Relationship Between Serum Testosterone Levels and Kidney Stones Prevalence in Men

**DOI:** 10.3389/fendo.2022.863675

**Published:** 2022-05-02

**Authors:** Fang Huang, Yongchao Li, Yu Cui, Zewu Zhu, Jinbo Chen, Feng Zeng, Yang Li, Zhiyong Chen, Hequn Chen

**Affiliations:** ^1^Department of Urology, Xiangya Hospital, Central South University, Changsha, China; ^2^National Clinical Research Center for Geriatric Disorders, Xiangya Hospital, Central South University, Changsha, China

**Keywords:** testosterone, kidney stones, prevalence, association, NHANES

## Abstract

**Background:**

The role of serum testosterone levels in male renal stone formation remains controversial. This study aimed to evaluate the relationship between serum testosterone levels and kidney stone prevalence in males.

**Methods:**

We conducted a cross-sectional study based on the data from the National Health and Nutrition Examination Survey 2011–2016, which included 6,633 male participants, to investigate the association between testosterone levels and the prevalence of kidney stones.

**Results:**

In this study, using the highest quartile of serum testosterone as a reference, a logistic regression model adjusted for confounders in all participants showed that the first quartile (OR: 1.375, p = 0.016), the second quartile (OR: 1.348, p = 0.021), and the third quartile (OR: 1.472, p = 0.003) of testosterone significantly increased kidney stone risks. In the 41–60 age group, the ORs of kidney stone risk in the first, second, and third of serum testosterone were 1.904 (P = 0.005), 1.599 (P = 0.040), and 1.734 (P = 0.015), respectively. This trend can also be found in the 61–80-year group, except in the first quartile of serum testosterone (OR: 1.169, P = 0.436). Adjusted smoothed curves suggest a non-linear relationship between the 8 quantiles of serum testosterone and the risk of kidney stones in all participants and the 61–80 age group and a significant negative relationship in the 41–60 age group (OR: 0.921, P = 0.0193). But no correlation was seen in the 20–40 group.

**Conclusions:**

Serum testosterone levels were significantly inversely associated with the prevalence of kidney stones in men over 40 years of age, but no correlation was seen in the 20–40 group. The role of testosterone in stone formation should be redefined, and its effect should be further verified.

## Introduction

There exists a persistent male predominance among urinary stone formers, with a two to three times higher prevalence compared with females (1, 2). Based on the gender differences observed in the ecological data, it makes sense to think that testosterone is the main cause. Several small human studies also reported higher levels of testosterone in men who formed stones than in a control group (3–6). Meanwhile, it has been reported that castration can significantly reduce the rate of stone formation in stone-induced ethylene glycol-fed rat models (7). Despite all that, the relationship between serum testosterone and urolithiasis in men remains controversial. First, it is more difficult to relate these findings to clinically significant associations between testosterone and urolithiasis because the associations were primarily established in small populations and those studies did not strictly control for confounding factors associated with testosterone and urolithiasis, such as obesity, hypertension, cardiovascular disease, diabetes, etc. Second, several studies have also suggested the opposite view, reporting an association between low serum testosterone levels and a higher incidence of urolithiasis (8, 9) or no relationship (10). By defining testosterone levels as low (≤300 ng/dl), normal (>300 ng/dl and <1,000 ng/dl), and high groups (≥1,000 ng/dl) based on a diagnosis of hypogonadism in men (11), Sirpi Nackeeran et al. analyzed the relationship between testosterone and the incidence of kidney stones in men using the data in the National Health and Nutrition Examination Survey (NHANES) database from 2013 to 2016, which showed no relationship between testosterone and kidney stones. But using the broad range of 300–1,000 ng/dl as the normal testosterone population to analyze the relationship with kidney stone incidence may obscure the underlying relationship (12). Third, metabolic syndrome and osteoporosis are known to be associated with a high incidence of stone formation (13–15) and low levels of testosterone (16, 17), which contradicts the prevailing view that high levels of testosterone can significantly promote stone formation. Fourth, as lifestyle-associated risk factors change, the susceptibility of men to urinary stones seems to be gradually decreasing compared with women. For example, a study reported a significant change in the male:female ratio of nephrolithiasis prevalence from 1.7:1 in 1997 to 1.3:1 in 2002 (18). Therefore, a large, representative cross-sectional study is needed to fully assess the relationship between serum testosterone and kidney stone prevalence in men. Thus, this study aimed to investigate an association between serum testosterone and kidney stone prevalence using representative data from the NHANES 2011–2016.

## Methods

### Study Design and Study Population

**Figure 1:**
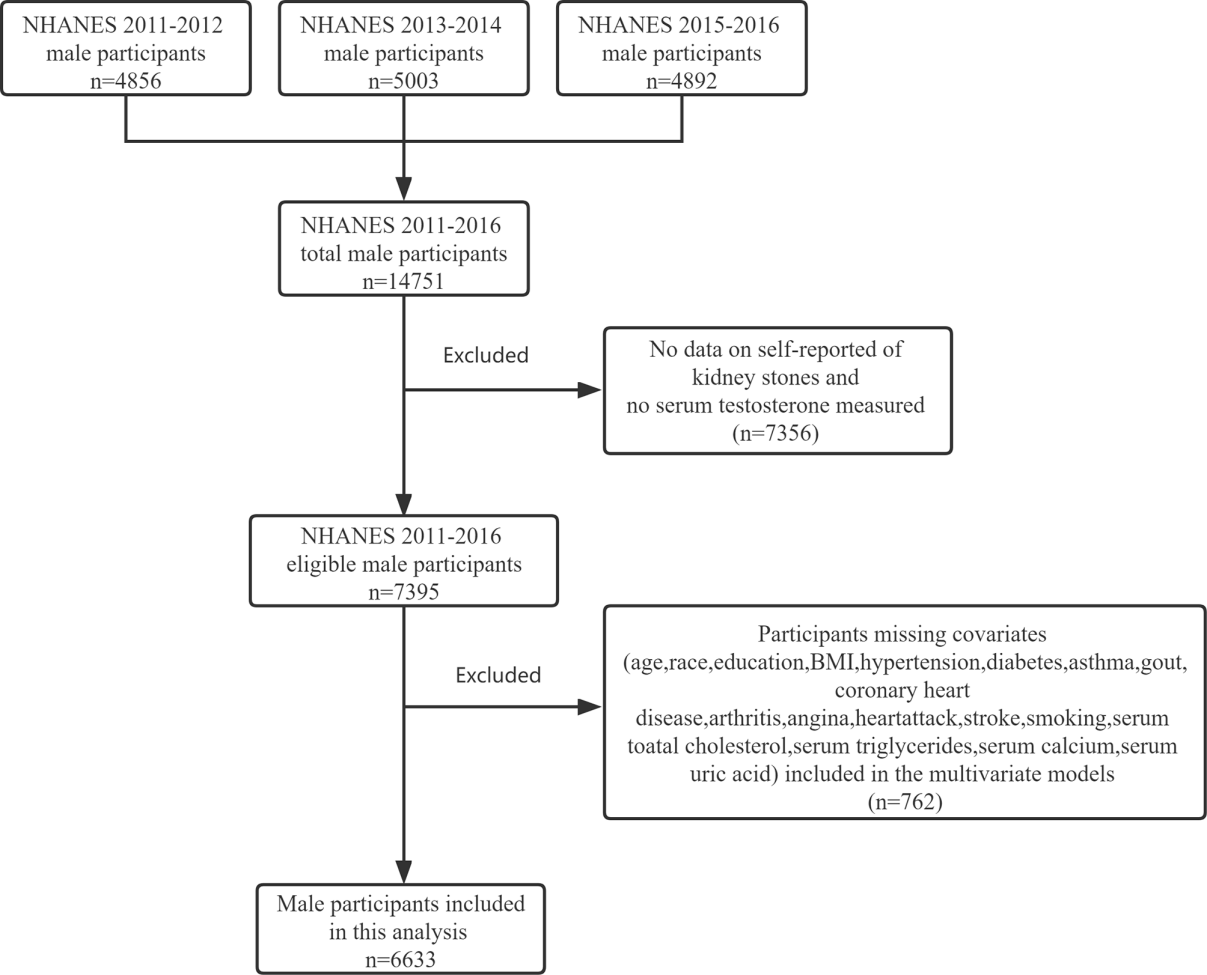
Flowchart identifying process of the NHANES 2011–2016 male participants inclusion and exclusion.

NHANES is a cross-sectional, nationally representative survey to collect health examination data using a stratified, multistage probability design to select a representative sample of the noninstitutionalized population of the United States. The data included health interviews, examination components, and laboratory tests administered by highly trained medical personnel. In this study, we included male participants who participated in the 2011–2012, 2013–2014, and 2015–2016 NHANES study cycles. The exclusion criteria are as follows (1): No data on self-reported kidney stones and no serum testosterone measured (2); Participants missing covariates, namely, age, race, education, BMI, hypertension, diabetes, asthma, gout, coronary heart, disease, arthritis, angina, heart attack, stroke, smoking, serum total cholesterol, triglycerides, calcium, and uric acid (see **Figure 1** for detailed information of inclusion/exclusion criteria). Eventually, 6633 participants were enrolled in the study.

### Variable Definitions and Data Collection

The dependent variable was kidney stones, which was defined by self-response to a question of a standardized questionnaire, “Have you ever had a kidney stone?” The serum total testosterone was measured using isotope dilution liquid chromatography-tandem mass spectrometry in the 2011–2012, 2013–2014, and 2015–2016 NHANES study cycles.

According to previous studies on factors affecting serum testosterone and the incidence of kidney stones, the following variables were included as covariates: age, race, education, body mass index (BMI), hypertension, diabetes, asthma, gout, coronary heart disease, arthritis, angina, heart attack, stroke, smoking, serum total cholesterol, triglycerides, calcium, and uric acid. The race was categorized as Mexican-American, non-Hispanic whites, non-Hispanic blacks, and Other/multicultural. According to completed years of schooling, education was categorized as ≤8 years, 9–12 years, and ≥12 years. BMI was calculated as the weight in kilograms divided by the height in meters squared, and obesity was defined as a BMI of 30 or higher. A history of gout and hypertension was obtained from the self-reported responses to questions asking whether a doctor had ever informed the respondent that they had the listed conditions. Participants were considered to have a history of smoking if they answered “Yes” to the question “Have you smoked at least 100 cigarettes in your entire life?” A history of diabetes was defined by self-response to the question, “Other than during pregnancy, have you ever been told by a doctor or health professional that you have diabetes or sugar diabetes?” Serum total cholesterol, triglycerides, calcium, and uric acid were obtained from the Standard Biochemistry Profile.

### Statistical Analysis

Initially, in the stone former and non-stone former groups, the chi-square test was used to analyze the proportion of categorical variables, and the Student’s t-test was used to analyze numerical variables. Previously reported factors affecting kidney stone prevalence, which included age, race, BMI, hypertension, diabetes, gout, coronary heart disease, arthritis, angina, heart attack, stroke, smoking, serum total cholesterol, triglycerides, calcium, and uric acid, were considered potential confounders and were incorporated into the stepwise forward multivariate logistic regression models to adjust the evaluation of the relationship between the quartile of serum testosterone and the prevalence of kidney stones.If serum testosterone was not eventually included in the stepwise forward multivariate logistic regression model, the enter logistic multivariate regression model (a model in which all the included independent variables are forced into the logistic regression equation in SPSS 22 software) was used. A test for linear trend was conducted with the use of quartiles of the total serum testosterone as a continuous variable.

We further applied a two-piecewise linear regression model to examine the threshold effect of the eight quantiles of serum testosterone on the prevalence of kidney stones using a smoothing function. The threshold level was determined using trial and error, including a selection of turning points along with a pre-defined interval and then choosing the turning point that gave the maximum model likelihood. A log-likelihood ratio test comparing the one-line linear regression model with a two-piecewise linear model was also conducted (19). This study was performed according to the guidelines of the NHANES and accounted for the complex survey design. All statistical analyses were performed with the statistical software EmpowerStats (http://www.empowerstats.com, X&Y Solutions, Inc., Boston, MA) and SPSS 22 for Windows (Chicago, IL, USA), taking NHANES sampling weights into account. A p-value of ≤0.05 (two-sided) was considered statistically significant.

## Results

Data were available on kidney stones and serum testosterone levels in 6,633 male adult participants. **Table 1** shows the characteristics of the study participants according to kidney stones. The prevalence of kidney stones was 10.4% in the population and participants were more likely to be obese, smoker, diabetes, hypertension, gout, arthritis, coronary disease, angina, heart attack, and stroke. Participants with kidney stones had significantly lower serum testosterone levels than those without stones (377.7 ± 175.7 vs 419.2 ± 187.1 ng/dl, p <0.001, **Table 1**), and the incidence of kidney stones decreased with the increase of testosterone quartile (p <0.001, **Table 1**).

**Table 1:** Characteristics of the study population, according to stone formers (n = 6,633).

	Non-stone formers(n = 5,945)	Stone formers(n = 688)	P*-*value^a^
Number of participants (%)	89.6	10.4	–
Age (years), mean ± SD	47.9 ± 17.5	57.6 ± 15.7	<0.001
Race/ethnicity (%)			<0.001
Mexican-American	13.7	13.2	
Non-Hispanic white	37.2	51.2	
Non-Hispanic black	22.3	12.4	
Other/multicultural	26.9	23.3	
Education (%)			0.667
≤8 years	9.7	10.8	
9–12 years	36.6	36.3	
>12 years	53.7	52.9	
BMI, mean ± SD (kg/m^2^)	28.4 ± 6.09	29.7 ± 6.05	<0.001
Hypertension (%)	33.6	50.0	<0.001
History of diabetes (%)	14.8	29.7	<0.001
History of asthma (%)	12.7	12.9	0.860
History of gout (%)	5.3	10.2	<0.001
History of arthritis (%)	18.5	33.7	<0.001
History of coronary heart disease (%)	4.1	11.5	<0.001
History of angina (%)	2.0	6.4	<0.001
History of heart attack (%)	4.2	10.2	<0.001
History of stroke (%)	3.1	4.9	0.017
History of high cholesterol level (%)	34.0	51.3	<0.001
Smoking>100	51.8	59.2	<0.001
Serum calcium, mean ± SD (mmol/l)	2.36 ± 0.09	2.34 ± 0.09	<0.001
Serum total cholesterol, mean ± SD (mg/dl)	4.87 ± 1.08	4.75 ± 1.11	0.005
Serum triglycerides, mean ± SD (mmol/l)	1.88 ± 1.54	2.05 ± 1.87	0.006
Serum uric acid, mean ± SD (mg/dl)	357.7 ± 77.2	357.7 ± 86.4	0.984
Serum total testosterone, mean ± SD (ng/dl)	419.2 ± 187.1	377.7 ± 175.7	<0.001
Testosterone quartile, % (range, ng/dl)			
Q1 (≤287.40)	24.3	31.5	<0.001
Q2 (287.41–386.00)	24.8	26.7	
Q3 (386.10–510.47)	25.0	25.1	
Q4 (>510.48)	26.0	16.6	

BMI, body mass index.

a Student's t-test was used to compare the differences of continuous variables, and Chi-square test was performed to compare the differences of categorical variables.

A strong negative correlation existed between serum testosterone quartiles and kidney stone history, with higher ORs of individuals reporting a stone history with lower testosterone quartiles in an unadjusted logistic regression model (**Table 2**). This trend is particularly pronounced in the 41–60 age group, and the OR value of the lowest serum testosterone quartile reporting a history of kidney stones was 2.391 times that of the highest serum testosterone quartile (P <0.001, **Table 2**). But it was statistically insignificant in the 20–40 age group (P = 0.178). The multivariate logistic regression analysis, controlling for factors known to impact kidney stones and serum testosterone, showed that the first quartile (OR 1.375, p = 0.016), the second quartile (OR 1.348, p = 0.021), and the third quartile (OR 1.472, p = 0.003) significantly increased the prevalence of kidney stones compared with the fourth quartile in all participants, but with P for trend equals 0.117 (**Table 2**). In subgroup analysis, using the highest quartile of serum testosterone as a reference in the 41–60 age group, the ORs of kidney stone risk in the first, second, and third of serum testosterone were 1.904 (95% CI: 1.212–2.991, P = 0.005), 1.599 (95% CI: 1.021–2.503, P = 0.040), and 1.734 (95% CI: 1.113–2.700, P = 0.015) and the trend test was statistically significant (P for trend = 0.001, **Table 2**). Although this trend was insignificant in the 61–80 group (P for trend = 0.966), the ORs for kidney stone risk were still higher in the second (OR: 1.471, 95% CI: 1.016–2.129, P = 0.041) and third (OR: 1.466, 95% CI: 1.020–2.106, P = 0.039) quartile of serum testosterone. However, the ORs of kidney stone risk in the first quartile of serum testosterone showed no significant difference compared to the highest quartile of serum testosterone in the 61–80 group (OR: 1.169, 95% CI: 0.789, 1.731, P = 0.436) (**Table 2**).

**Table 2:** The association of the prevalence of kidney stones and testosterone evaluated by logistic regression analysis in subgroups stratified by age.

	Unadjusted, OR (95% CI)	P-value	Adjusted ^b^, OR (95% CI)	P-value
All, Testosterone^a^				
Q1 (≤287.40)	2.04 (1.607–2.585)	<0.001	1.375 (1.061–1.781)	0.016
Q2 (287.41–386.00)	1.691 (1.324–2.158)	<0.001	1.348 (1.041–1.739)	0.021
Q3 (386.10–510.47)	1.578 (1.233–2.020)	<0.001	1.472 (1.144–1.895)	0.003
Q4 (>510.48)	reference		reference	
P for trend		<0.001	0.117	
Age (20–40), Testosterone^a^				
Q1 (≤313.69)	1.531 (0.920–2.549)	0.101	–	–
Q2 (313.7–418.0)	1.038 (0.599–1.801)	0.893	–	–
Q3 (418.1–542.0)	1.282 (0.757–2.171)	0.355	–	–
Q4 (>542.01)	reference		–	–
P for trend		0.178		
Age (41–60), Testosterone^a^				
Q1 (≤280.31)	2.391 (1.565–3.654)	<0.001	1.904 (1.212–2.991)	0.005
Q2 (280.31–370.00)	1.785 (1.149–2.775)	0.010	1.599 (1.021–2.503)	0.040
Q3 (370.10–492.22)	1.827 (1.177–2.836)	0.007	1.734 (1.113–2.700)	0.015
Q4 (>492.23)	reference		reference	
P for trend		<0.001		0.001
Age (61–80), Testosterone^a^				
Q1 (≤268.0)	1.613 (1.134–2.296)	0.008	1.169 (0.789–1.731)	0.436
Q2 (268.1–360.9)	1.776 (1.252–2.520)	0.001	1.471 (1.016–2.129)	0.041
Q3 (361.0–492.0)	1.582 (1.110–2.254)	0.011	1.466 (1.020–2.106)	0.039
Q4 (>492.1)	reference		reference	
P for trend		0.008		0.966

CI, confidence interval; HR, hazard ratio.

a Presented in quartiles (ng/dl).

b Adjusted for age, race, BMI, hypertension, diabetes, gout, coronary heart, disease, arthritis, angina, heart attack, stroke, smoking, serum total cholesterol, triglycerides, calcium, and uric acid.

**Figure 2:**
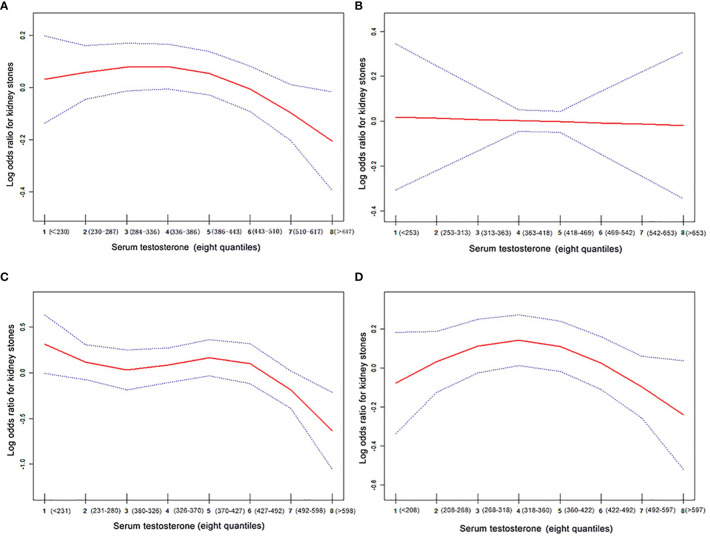
The association curve between serum testosterone levels and log odds for kidney stones. The solid red line represents the smooth curve fit between variables. The blue line represents the 95% of confidence interval from the fit. The values have been adjusted for age, race, BMI, hypertension, diabetes, gout, coronary heart, disease, arthritis, angina, heart attack, stroke, smoking, serum total cholesterol, triglycerides, calcium, and uric acid. **(A)** All participants; **(B)** the 20–40 age group; **(C)** the 41–60 age group, and **(D)** the 61–80 age group.

Adjusted smoothed curves suggest a non-linear relationship between the eight quantiles of serum testosterone and the risk of kidney stones (**Figures 2A, D**; **Table 3**) in all participants and the 61–80 age group, with a significant negative relationship in the 41–60 age group (**Figure 2C**, OR: 0.921, 95% CI: 0.860–0.987, P = 0.0193, **Table 3**). But no correlation was seen in the 20–40 age group (**Figure 2B**; **Table 3**). Using a two-piecewise regression adjusted model to evaluate the relationship between the 8 quantiles of serum testosterone and the risk of kidney stones, we found that the inflection point was Q5 (360–422 ng/dl) in all participants. When the serum testosterone ≥Q5, there was a significant negative correlation between serum testosterone and kidney stone risks (OR = 0.875, 95% CI: 0.731 to 0.981, p = 0.01). By contrast, there was no correlation when the serum testosterone<Q5 (**Table 3**). This trend was also found in the 61–80 age group. Serum testosterone exhibited a negative correlation with kidney stone risk when ≥Q5 (360–422 ng/dl) (OR = 0.831, 95% CI: 0.717–0.963, P = 0.014). When serum testosterone is less than the inflection point, there is no significant relationship (**Table 3**). In the 41–60 age group of the two-piecewise regression adjusted model, when testosterone ≥Q6 (427–492 ng/dl) (OR = 0.657, 95% CI: 0.487–0.886, P = 0.006), the negative correlation between serum testosterone and kidney stone risk was more significant than in Model I one-line (**Table 3**).

**Table 3:** Threshold effect analysis of testosterone on the prevalence of kidney stones using piece-wise linear regression.

	Crude OR^b^ (95% CI)	p*-*value		Adjusted OR^c^ (95% CI)	p-value
All, Testosterone^a^					
Model I one-line	0.904 (0.873, 0.937)	<0.001		0.972 (0.935, 1.011)	0.159
Model II turning point: Q5 (386–443)			Model II turning point: Q5 (386–443)		
<Q5	0.942 (0.884, 1.002)	0.059	<Q5	1.033 (0.966, 1.105)	0.336
≥Q5	0.843 (0.764, 0.930)	<0.001	≥Q5	0.875 (0.731, 0.981)	0.010
Age (20–40) Testosterone^a^					
Model I one-line	0.953 (0.880, 1.031)	0.2271		0.995 (0.906, 1.092)	0.9118
Model II turning point: Q3 (313–363)			Model II turning point: Q3 (313–363)		
<Q3	0.807 (0.603, 1.080)	0.1499	<Q3	0.809 (0.598, 1.096)	0.1721
≥Q3	1.006 (0.891, 1.136)	0.9257	≥Q3	1.063 (0.932, 1.213)	0.3645
Age 41–60 Testosterone^a^					
Model I one-line	0.892 (0.839, 0.948)	<0.001		0.921 (0.860, 0.987)	0.0193
Model II turning point: Q5 (370–427)			Model II turning point: Q6 (427–492)		
<Q5	0.941 (0.844, 1.049)	0.2736	<Q6	0.995 (0.906, 1.092)	0.9084
≥Q5	0.810 (0.680, 0.964)	0.0179	≥Q6	0.657 (0.487, 0.886)	0.006
Age 61–80 Testosterone^a^					
Model I one-line	0.930 (0.883, 0.979)	0.005		0.979 (0.924, 1.038)	0.483
Model II turning point: Q3 (268–318)			Model II turning point: Q5 (360–422)		
<Q3	1.141 (0.938, 1.389)	0.187	<Q5	1.084 (0.979, 1.200)	0.120
≥Q3	0.872 (0.805, 0.943)	<0.001	≥Q5	0.831 (0.717, 0.963)	0.014

CI, confidence interval; HR, hazard ratio.

a Presented in eight quantile (ng/dl) and was conducted the with eight quantiles of the total serum testosterone as a continuous variable.

b Adjusted for: none.

c Adjusted for: age, race, BMI, hypertension, diabetes, gout, coronary heart, disease, arthritis, angina, heart attack, stroke, smoking, serum total cholesterol, triglycerides, calcium, and uric acid.

## Discussion

The relationship between sex hormones and urolithiasis has been widely discussed by urologists because of the gender difference in the incidence of urolithiasis (2). Most scholars believe that testosterone is the main reason for this observational data, but they seem to ignore the role of estrogen in the regulation of urolithiasis in women (1, 7, 20). Furthermore, whether androgens promote stone formation is still a controversial topic and lacks a large, representative cohort study (9).

Using a large cross-sectional study, we examined the association between serum testosterone and kidney stones in men. For all participants, the mean serum testosterone of patients with stones was significantly lower than that of normal participants, and in an unadjusted logistic regression model, the prevalence of kidney stones was inversely associated with the quartile of serum testosterone. Furthermore, in multivariate logistic regression, we adjusted for confounders, and the OR values of the first three quartiles involved in kidney stone risk were still 1.3–1.4 times that of the highest quartile, which was statistically significant. Notably, there is a nonlinear relationship between the eight quantiles of serum testosterone and the risk of kidney stones after fully adjusting for confounders. When the serum testosterone ≥Q5 (360–422 ng/dl), there was a significant negative correlation between serum testosterone and kidney stone risks. By contrast, there was no correlation when the serum testosterone <Q5.

In the introduction, we mentioned the NHANES (2013–2016) study by Sirpi Nackeeran et al. which showed that testosterone was not related to the incidence of kidney stones in men (12). Their results differ from ours because they grouped testosterone levels differently from our study. They classified testosterone into low (≤300 ng/dl), normal (>300 ng/dl and <1,000 ng/dl), and high groups high (≥1,000 ng/dl) based on diagnosis of hypogonadism in men (11). Their normal testosterone group almost covered the range of testosterone levels in the Q2–Q4 groups (**Tables 1**, **2**) in our study. Therefore, their findings may reflect only the relationship between male hypogonadism and kidney stone incidence, ignoring the potential association between different testosterone levels and kidney stone incidence. In our study, we added the data from the NHANES from 2011 to 2012 and adopted a more scientific quartile grouping method for testosterone to analyze the relationship between different testosterone levels and the incidence of male kidney stones. Therefore, our results may better reflect the potential relationship between testosterone levels and the incidence of kidney stones in men.

The results of our study seem to be inconsistent with the prevailing view, testosterone promotes stone formation, which is mainly based on small sample size studies (3–5) and some observations that men have a higher risk of developing kidney stones than women (2). As we mentioned before, explaining this sex difference in kidney stone incidence with androgens seems to ignore the protective effect of estrogen on stone formation in women. Furthermore, some studies have found that the incidence of kidney stones is lower in women than in men before age 50, but the gender difference in incidence narrows in people aged >50 years, suggesting that estrogen may be the main cause of gender differences in kidney stones (1, 21). In the rat ethylene glycol model of urolithiasis studies also have shown that testosterone can promote hyperoxaluria caused by increasing liver glycolate oxidase (GAO) levels, which results in calcium oxalate crystal deposition in the renal interstitium (22, 23). As far as we know, the rat ethylene glycol model of urolithiasis relies on artificial mechanisms that are not comparable with the pathophysiology of naturally occurring stone disease in humans (24). *Via* ingestion of ethylene glycol, it causes intratubular calcium oxalate (CaOx) crystallization and, in the setting of renal injury, can lead to discrete crystal formation in the renal interstitium (24, 25). However, in natural human stone formation, no evidence exists to show that stones form secondary to oxalate-induced renal injury and calcium oxalate crystals are deposited in the renal interstitium (24, 26, 27). Several studies have also reported a higher incidence of urolithiasis in men with low serum testosterone levels (8, 9). Although the results of our large, representative cross-sectional study are consistent with these smaller studies and are consistent with observations of high stone incidence and low testosterone levels in diseases closely associated with stones, such as metabolic syndrome, we cannot draw arbitrary conclusions at this time.

Interestingly, in subgroup analysis, after adjusting for confounders, we found no association between serum testosterone levels and stone incidence in the 20–40 age group, but a significant negative association in the 41–60 age group. Meanwhile, in the 61–80 group, serum testosterone exhibited a negative correlation with kidney stone risk when ≥Q5 (360–422 ng/dl). This result is consistent with the physiological cycle of male testosterone levels, which decline with age, gradually decreasing with each decade after the age of 40 years (28). There are a large number of comorbidities in men associated with low serum testosterone, namely, heart failure, vascular disease, osteoporosis, dyslipidemia, type 2 diabetes, metabolic syndrome, and obesity (28, 29), which are significantly associated with a higher incidence of kidney stones (13, 14, 30). Therefore, we have reason to believe that there is a direct or indirect relationship between testosterone and the occurrence of urinary stones in men after 40 years of age. In men aged 20–40 years, most individuals have normal gonadal function, so testosterone is in a stable balance, and the regulation of physiological metabolism is also in a stable state. Therefore, testosterone does not appear to be associated with kidney stone development in this age group. When men are over 40 years old, some gonadal dysfunctions cause a significant decrease in testosterone release and physiological metabolism disorders, resulting in metabolic diseases and kidney stones. Thus, testosterone in men over 40 years old shows the same protective effect on the occurrence of urinary stones as estrogen in postmenopausal women (1, 31). Testosterone levels were inversely associated with kidney stone incidence in men over 40 years of age, which may be associated with testosterone-related metabolic diseases such as hypertension, diabetes, and metabolic syndrome. But because the role of testosterone in human metabolism is too complex to be understood in a cross-sectional national design, further research is needed in the future.

The current large sample cross-sectional national study provided evidence that serum testosterone levels are associated with kidney stone risk and that testosterone may have a protective effect on kidney stones in men older than 40. According to rigorous sampling design, high-quality research measurements, and detailed quality control procedures, the NHANES selected representative populations from the national population of the United States for the study, which made our research conclusions more representative. At the same time, we adjusted for confounders that might be responsible, and fully demonstrated an independent correlation between testosterone levels and the prevalence of kidney stones. There are a lot of limitations in our study. Most importantly, this is a cross-sectional observational study, and the fundamental flaw is that it can only assess correlation, not causation; Secondly, as self-reported data based on questionnaires are used in this study, the results are easily misclassified; Third, although we strictly adjusted multiple covariates, our findings might have been affected by residual confounding factors. Fourth, as mentioned before, some studies suggest that estrogen may play a role in the development of kidney stones in women. But in the incidence of kidney stones in men, one study suggested that estrogen may not play a role in stone formation (12). Since estrogen was not included in the NHANES Database from 2011 to 2012, this study did not include estrogen for analysis.

## Conclusion

Serum testosterone levels were significantly inversely associated with the prevalence of kidney stones in men over 40 years of age, but no correlation was seen in the 20–40 age group. The role of testosterone in stone formation should be redefined and its effect should be further verified.

## Data Availability Statement

The datasets presented in this study can be found in online repositories. The names of the repository/repositories and accession number(s) can be found below: https://www.cdc.gov/nchs/nhanes/.

## Ethics Statement

The studies involving human participants were reviewed and approved by The Wake Forest School of Medicine Institutional Review Board deemed this study of publically available, de-identified data exempt from human subjects research. The patients/participants provided their written informed consent to participate in this study.

## Author Contributions

FH: Conceptualization, Visualization, Methodology, Writing original draft. YoL: Investigation, Resources. YC: Validation, Data curation, Investigation. ZZ: Investigation, Resources. JC: Writing review & editing. FZ: Investigation, Resources. YaL: Methodology, Software, Formal analysis. ZC: Methodology, Software, Formal analysis. HC: Conceptualization, Project administration, Supervision. All authors listed have made a substantial, direct, and intellectual contribution to the work and approved it for publication.

## Funding

This study was supported by the National Natural Science Foundation of China (82170781).

## Conflict of Interest

The authors declare that the research was conducted in the absence of any commercial or financial relationships that could be construed as a potential conflict of interest.

## Publisher’s Note

All claims expressed in this article are solely those of the authors and do not necessarily represent those of their affiliated organizations, or those of the publisher, the editors and the reviewers. Any product that may be evaluated in this article, or claim that may be made by its manufacturer, is not guaranteed or endorsed by the publisher.
